# Timing and context of dolphin clicks during and after mine simulator detection and marking in the open ocean

**DOI:** 10.1242/bio.031625

**Published:** 2018-02-15

**Authors:** Sam H. Ridgway, Dianna S. Dibble, Jaime A. Kennemer

**Affiliations:** 1 Neurobiology Group, National Marine Mammal Foundation, 2240 Shelter Island Drive #200, San Diego, CA 92106, USA; 2 Department of Pathology, School of Medicine, University of California, San Diego, 9500 Gilman Drive, La Jolla, CA 92093, USA; 3 U.S. Navy Marine Mammal Program, Space and Naval Warfare Systems Center San Diego 53560 Hull Street, San Diego, CA 92152 , USA

**Keywords:** Dolphin, Clicks, Click packets, Victory squeal, Mine simulators, Cameras

## Abstract

Two dolphins carrying cameras swam in the ocean as they searched for and marked mine simulators – buried, proud or moored. As the animals swam ahead of a boat they searched the ocean. Cameras on their harness recorded continuous sound and video. Once a target was detected, the dolphins received a marker to take to the simulator's location. During search and detection, dolphins made almost continuous trains of varying interval clicks. During the marking phase, shorter click trains were interrupted by periods of silence. As the dolphins marked simulators, they often produced victory squeals – pulse bursts that vary in duration, peak frequency and amplitude. Victory squeals were produced on 72% of marks. Sometimes after marking, or at other times during their long swims, the dolphins produced click packets. Packets typically consisted of two to 10 clicks with inter-click intervals of 7-117 ms followed by a silence of 223-983 ms. Click packets appeared unrelated with searching or marking. We suggest that the packets were used to improve signal to noise ratios for locating a boat or other distant object. Victory squeals produced when marking the targets suggest to us that the dolphins know when they have succeeded in this multipart task.

## Abstract (precis)

**Summary:** Dolphins wore cameras so we could hear them and watch them mark mine simulators. We observed rhythmic click trains, victory squeals, and click packets with their behavioral context.

## INTRODUCTION

For more than 50 years, *Tursiops truncatus* have aided human operators' ocean searches. For the first time we were able to put cameras on dolphins cooperating with us in the open ocean. They searched for, detected, and marked mine simulators. The cameras on the dolphins allowed us to hear all their sounds, simultaneous with video of their behavior. Thus, we can report what each dolphin was doing when these sounds occurred.

There are several research papers on detection of mine-like targets in San Diego Bay using acoustic recording devices carried by the dolphin (Martin et al., 2003, 2005; Houser et al., 2005; Au and Martin, 2012). This previous work employed dedicated research dolphins in shallow waters within San Diego Bay. Operational working dolphins that must be skilled at finding real enemy mines (Renwick et al., 1997; Myers, 2015) interact with people daily in detecting practice moored, buried and proud sea floor-simulated mines. These dolphins swim in various locations in the ocean. We placed cameras on two such dolphins as the animals practiced in the Pacific Ocean. These video and sound recordings add to our understanding about dolphin behavior and sound as they complete these tasks.

Experiments over the past 60 years have shown that dolphins produce trains of sonar clicks and hear returning echoes for target detection (Kellogg, 1958; Norris et al., 1961; Morozov et al., 1972; Au et al., 1974). Usually, time intervals between successive clicks (ICI) in a click train are between 10 and 160 ms, depending on target distance (Morozov et al., 1972; Au et al., 1974). The ICIs are longer than the two-way travel time (TWT) – the time needed for a click to travel from the dolphin to the target and back (Murchison, 1980; Au et al., 1982; Kadane and Penner, 1983). Thus, the following click in the dolphin's click train occurred after the preceding click's echo returned to the animal. In these earlier works, the targets were approximately 100 m or less away and the researchers did not report click packets from the dolphins.

A different click strategy was first mentioned in a brief note by Ivanov and Popov (1978) with targets at a greater distance. They noted that *T. truncatus* in a detection task produced small ‘groups’ of clicks separated by silent periods. These groups of clicks or packets of clicks had ICIs much shorter than the round-trip acoustic propagation to and from the target. These packets of clicks were observed when dolphins reported targets over 140 m away. Click packet use in this species was discussed more thoroughly by Ivanov (2004). The click packets consisted of several clicks in succession followed by a period of silence that was at least twice as long as the TWT. Using click packets, dolphins detected targets over 650 m away (Ivanov, 2004).

Click packets were also recorded from free-ranging delphinids, *Pseudorca crassidens* and *Grampus griseus.* Both of these species are pelagic and are normally found far off-shore in deep water. They hunt various types of fish and cephalopods (Madsen et al., 2004). Madsen et al. came to no conclusion about why these delphinids sometimes employed click packets. Rankin et al. (2015) observed 110 click packets from free ranging groups of *Steno bredanensis* in the Pacific Ocean. They noted that these click packets were temporally distinct from typical click trains. Neither of these observations of delphinids in the wild identified click packet targets or the range of the targets.

Click packets targets were known only for stationary dolphins (Ivanov, 2004; Finneran, 2013). Ivanov (2004) noted that dolphins used click packets when targets were over 140 m away from the animal's station. Finneran (2013) tested three *T. truncatus* using a phantom echo generator (PEG). He presented echo returns representing 25-800 m in target distance and found that two of his three dolphin subjects changed strategies from producing click trains to click packets when the echo delay represented a target distance over 75 m. Finneran (2013) and Finneran et al. (2014) observed that the ICI within each packet is well below the TWT, but the period of silence between packets was much greater than the TWT, suggesting that the animals were waiting for the packet of echoes to return before producing another click packet. Here, we define a click packet using findings from Finneran et al. (2014) as a group of clicks followed by a period of silence of 200 ms or more. This period of silence suggests that the dolphin is listening for echoes returning from objects at least 150 m away.

In addition to continuous click trains and click packets, we also observed rapid click bursts. Previously we have shown the time amplitude and frequency spectrum of these rapid click bursts (Ridgway et al., 2014, 2015). For many years we have heard these rapid bursts of clicks that vary in duration, peak frequency and amplitude that we call a victory squeal (VS). The VS is often heard when the dolphin seizes a fish (Ridgway et al., 2015). During training this click burst occurs after a trainer's bridge or whistle confirming a correct response (Ridgway et al., 2014). Thus, the VS is associated with food and with expectation of food reward (Ridgway et al., 2014, 2015). We have recently shown that the VS is often produced at the instant a task is completed (Dibble et al., 2016). Like the VS produced when a fish is captured and also after a trainer's bridge, the VS produced at the instant of task completion suggests an emotional expression, possibly induced by dopamine release, indicating that dolphins recognize that their task is completed. We wanted to see if the two experienced dolphins demonstrated this emotional VS the instant they marked the mine simulator.

**Fig. 1.**
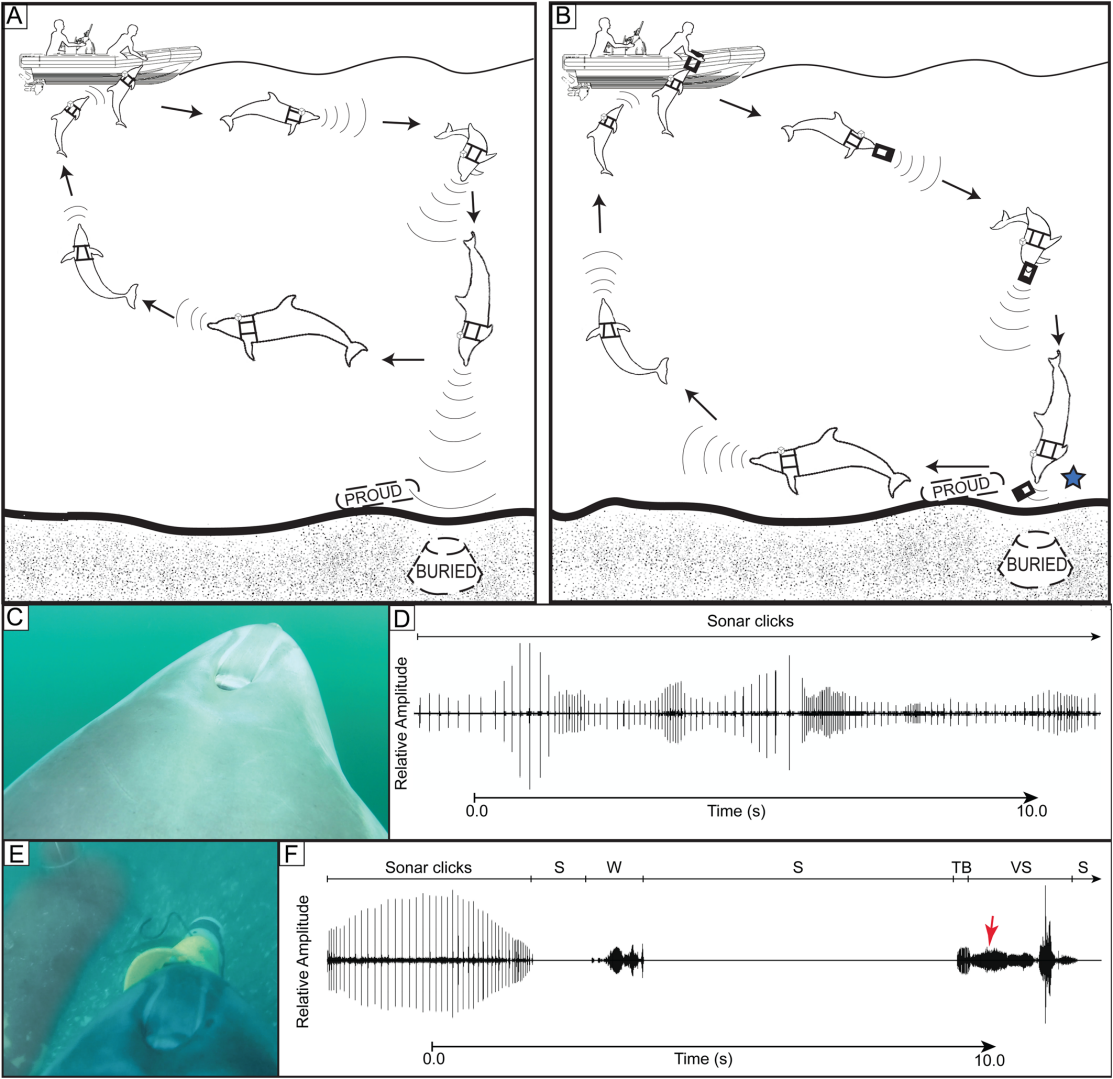
**Schematic illustration of dolphins hunting for and marking buried and proud sea floor mine simulator.** (A) A schematic representation of the first two tasks, showing a dolphin searching for and detecting a sea floor mine simulator and returning to the boat to confirm detection. (B) Schematic representation of the dolphin receiving a marker, swimming down to the sea floor, placing the marker near the mine simulator and returning to the operators' boat, completing the multipart task. During the actual task, the animal and boat may move over much longer distances than represented. Often, during searching and marking, the animal may be several hundred meters away from the operators' boat and unseen at depth. A star symbol indicates where the VS production begins as the dolphin releases the marker. (C) A view from the camera attached dorsally showing the dolphin's back, blowhole and forehead as the animal swims. The view forward of the animals varied from one to several meters depending on depth and water clarity. (D) Trains of clicks vary widely in amplitude and repetition rate as the dolphin searches for the target. (E) View from the dorsally mounted camera as the dolphin marks a proud mine simulator. (F) Relative sound amplitude as the dolphin nears the target. Trains of sonar clicks, a whistle (W), periods of silence (S) terminate in a in a brief terminal buzz (TB) and VS as the dolphin marks a proud mine simulator. The red arrow indicates when the dolphins mark based on the view in panel E. Since the sonar clicks terminated about 8 s before marker release, vision may have been more important for marker placement in this case.

**Fig. 2.**
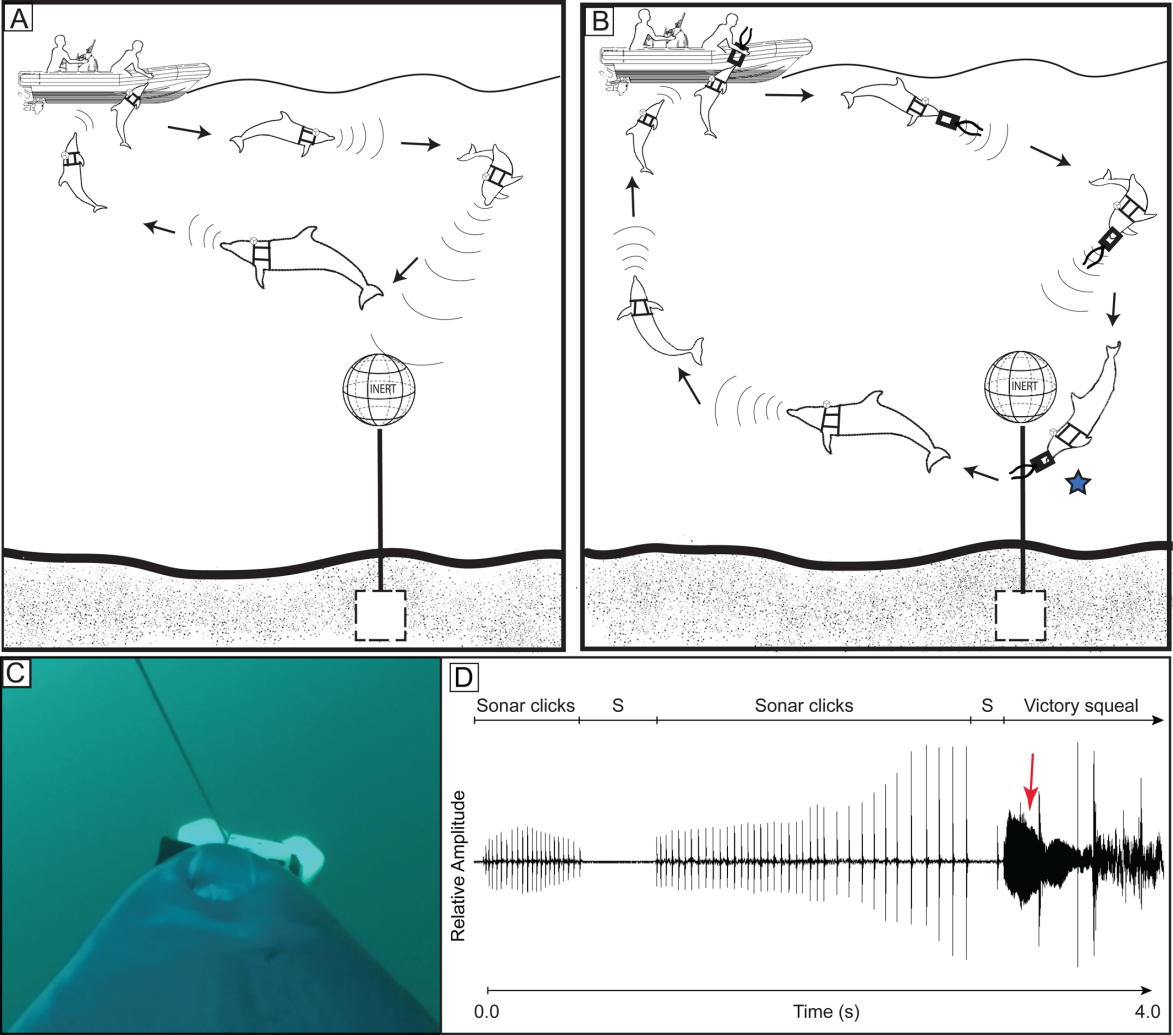
**Schematic illustration of a dolphin hunting for and marking a moored mine simulator.** (A) A schematic representation of a dolphin searching for and detecting a sea floor mine simulator and returning to the operators' boat to confirm detection. (B) Schematic representation of the dolphin receiving a marker, swimming down, attaching the marker to the simulated mine cable and returning to the operators' boat, completing the task. During the actual task, the animal and boat may move over much longer distances than represented. Often, during searching and marking, the animal may be several hundred meters away from the operators' boat and unseen at depth. A star symbol indicates where VS production typically begins. (C) View from the dorsally mounted camera as the dolphin attached a marker to a mooring cable. (D) Relative sound amplitude as the dolphin nears the target. Trains of sonar clicks (SC) interrupted by periods of silence (S) terminate in a VS as the dolphin secures (see red arrow) the marker to the cable.

We wanted to link behavior and sounds of dolphins completing tasks in the open ocean. During the tasks dolphins first swam in front of the operators' boat searching. On detection of a mine simulator, each animal returned to their operator to receive a marker (Figs 1 and 2). They placed the marker near the bottom mine simulator or latched the marker to the cable of the moored mine simulator (Fig. 2). The dolphins then returned to the operators' boat to be rewarded with food. During the entire time, cameras on the dolphins' harnesses recorded video and sound.

## RESULTS

From the recordings of the harness mounted cameras, we could see the dolphin's head and rostrum as moved from side to side in typical scanning motions during the search. We could see the movements of the dolphin's nasal plug as it produced clicks and other sounds. During the search and detection phase (Figs 1A and 2A), both dolphins typically produced continuous trains of clicks. During the marking phase, the dolphins produced trains of clicks that were much shorter in duration and the trains were frequently interrupted by periods of silence ending in a VS at marking (Figs 1 and 2, Table 1).

**Table 1.**
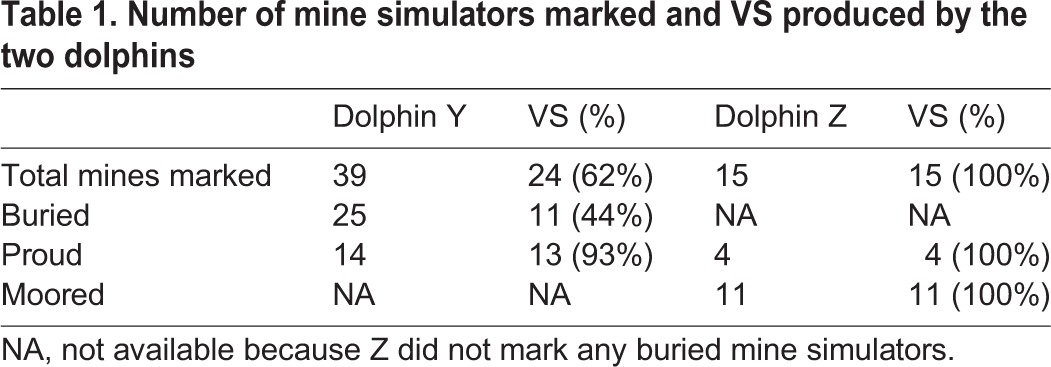
**Number of mine simulators marked and VS produced by the two dolphins**

During 148 min of recordings, dolphin Y detected and marked 25 buried mine simulators. She detected and marked (Fig. 1C) 14 proud mine simulators. On these 39 detections Y reported positive target detection and took a marker from the boat operator. The dolphin placed the practice marker near the previously detected simulator (Fig. 1B). On 24 (62%) of total marks dolphin Y produced a VS while placing the marker (Fig. 1C,D, Table 1). On 13 (93%) marks for proud mine simulators a VS was produced (i.e. Fig. 1E,F, Table 1). However, on only 44% of marks of buried mine simulators was a VS produced.

During 190 min of recording, dolphin Z marked four proud simulators and produced a VS every time. Dolphin Z also detected and marked 11 moored simulators (Fig. 2A,B,C), producing a VS each time (Fig. 2, Table 1). During searches, click trains from dolphin Z were similar to those of dolphin Y (Fig. 1D).

**Fig. 3.**
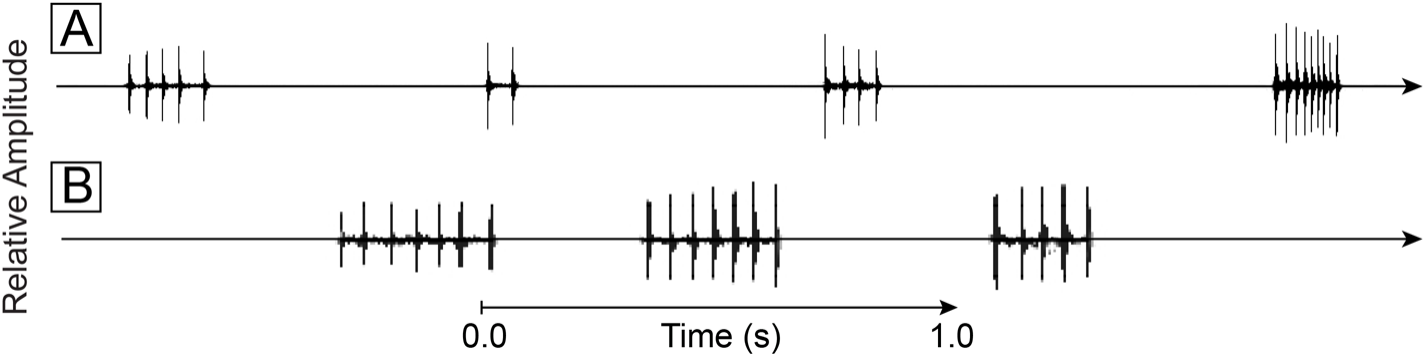
**Click packets and click packet series produced by dolphins after marking mine simulators and swimming near the sea floor during their return to the operators' boat.** (A) Dolphin Z; (B) dolphin Y.

Occasionally, the dolphins stopped producing trains of clicks, and after a period of silence, produced a click packet(s) (Movies 1 and 2). The majority of click packets occurred when the dolphin was returning to the boat after marking. One-hundred and six individual packets were recorded, creating 38 packet series. A lone click packet was often recorded from dolphin Z. On average click packet series from both dolphins consisted of about three individual packets in succession (Fig. 3). The number of clicks within a click packet varied from two to 14, but on average packets consisted of six clicks followed by a period of silence lasting 223-983 ms (Table 2). Inter-click interval within packets averaged 32.9 ms, but varied considerably from 7 to 117 ms. The period of silence between packets averaged 450 ms with a median of 364 ms. We assume that during this period of silence the dolphin listens for distant echoes to return. The average and median period of silence suggest that the dolphins were assessing targets about 273-338 m distant. The maximum inter-packet interval would suggest targets over 700 m distant.

**Table 2.**
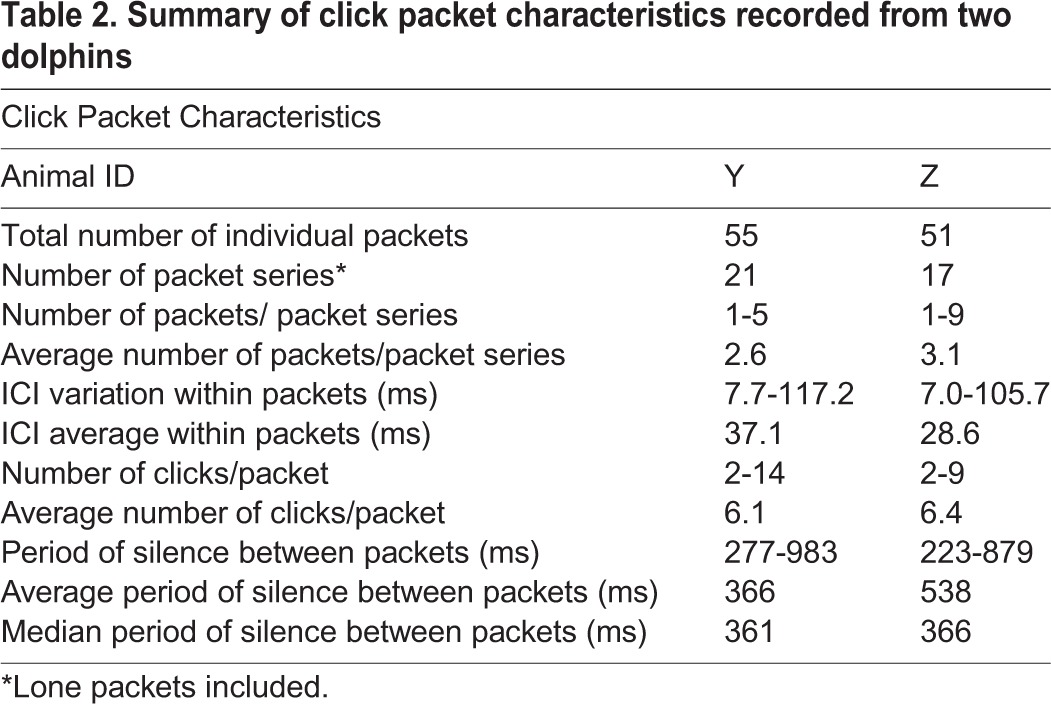
**Summary of click packet characteristics recorded from two dolphins**

Packets did not appear to be related to the mine-hunting simulator detection task. Since the majority of click packets occurred after the dolphin marked a mine simulator, the animal was likely assessing something of interest in the distance, possibly on the surface of the ocean. This was suggested during video review of the dolphin's head-scanning motions as well as when click packets occurred after marking. For example, 18 packet series occurred after a successful mark when the dolphin was swimming along the bottom and beginning its return to the boat. Fifteen packet series occurred as the animal swam along the ocean surface next to the boat. Only five packet series occurred as the animal descended toward the sea floor before marking a simulator.

## DISCUSSION

For the first time, simultaneous sounds and behavior were recorded as dolphins searched in the open ocean. While the dolphins search, detect, and finally mark targets they are out of sight underwater. They are often well away from their operators, perhaps several hundred meters away at times. Without cameras on dolphins, it has not been possible to observe their behavior during these tasks. However, when dolphins wear cameras, their sounds and behavior can be linked. The majority of sounds produced during the search and detection phase (Figs 1A and 2A) were continuous click trains (Fig. 1D). During this phase, the mine simulators were detected when the dolphins were some distance away from the mine simulators. Most often, the animals did not swim all the way down to the sea floor to detect the simulator. During the marking phase click timing was different. Both dolphins produced click trains that were significantly shorter in duration that were interrupted by periods of silence. As the markers were being placed (Figs 1C,E and 2C) the dolphins often produced a VS (Figs 1D,F and 2D, Table 1). At times the dolphins whistled simultaneously while clicking and they occasionally whistled during periods of silence. Conspicuously, click packets occasionally occurred – several clicks in a row followed by a period of silence (Fig. 3).

The dolphin's click packets may be an attempt to recognize a distant target of interest. For example, when the dolphin is far away from the operators' boat and swimming along the sea floor, packets may have been used to locate the boat on the surface. The dolphin must return to the operator to receive its fish reward for task completion. Perhaps click packets allowed the dolphin to find the shortest route to the boat. These observations are consistent with previous findings on dolphin click packet use. The investigations of Ivanov (2004) and Finneran (2013) found that stationary dolphins locating targets over a range of distances used packets for the more distant targets.

Packet use has been suggested previously for detection of surface targets. A trained beluga (*Delphinapterus leucas*) used packets. This beluga detected targets within the test range in Kaneohe Bay mentioned earlier (Au et al., 1974; Murchison, 1980; Kadane and Penner, 1983). The beluga sometimes produced packets of four or five clicks with an ICI of around 40 ms, which was shorter than the TWT to the target (Turl and Penner, 1989). At the time, click packet use had not been observed when *T. truncatus* detected targets over the same range in Kaneohe Bay, Hawaii. In their natural habitat, belugas spend part of each year inside Arctic pack ice. Au (1993) speculated that the beluga's packet use might relate to unknown adaptations to a life in an ice-covered habitat. Indeed, belugas sometimes live in ice covered sea with only small openings to the air where they must breath. Belugas may dive deep to feed (Ridgway et al., 1984; Martin and Smith, 1992) on or near the bottom, hundreds of meters down. In order to survive, belugas must rapidly locate these surface openings after foraging at great depth.

Click packets of belugas or dolphins seem ideal for recognizing distant targets in an ocean of background noise. Dolphins hear and their brain responds to their own clicks (Bullock and Ridgway, 1972). A dolphin must know acoustic features of its clicks and how many clicks it produces. In essence, when the dolphin produces a packet, it produces a coded signal that can be matched with the echo return. These types of matched filters have been suggested previously by Au and Martin (1989) and by Leighton et al. (2012).

Another recognizable sound often occurred. We heard this sound when the dolphin marked a mine simulator. This sound was a burst of pulses that varied in duration, peak frequency and amplitude – the VS. This VS has been associated with fish capture and with expectation of a reward after a trainer's bridge for correct behavior (Ridgway et al., 2014, 2015; Dibble et al., 2016). When dolphins hunt, they locate prey with clicks, refine the ‘image’ when they near the prey by transitioning clicks into a terminal buzz and finally produce a victory squeal at prey capture (Ridgway et al., 2015; Harder et al., 2016). Recently, we have reported that dolphins produce a VS after completion of task components, without a trainer's bridge (Dibble et al., 2016). Dolphin Z produced a VS every time she marked a target. However, since Y could not visually or tactilely confirm the buried simulators when marking the target, perhaps the VS was produced less often because she was less certain of her success.

Because the VS was produced so often as the dolphin marked, we considered it as a sign that the dolphin recognized when its multipart task was completed. The VS is also a sign that the animal expects reward. Reward expectation has been connected to a dopamine release in the brain. Early brain stimulation studies demonstrated consistent timing that may link the VS with brain dopamine release (Ridgway et al., 2014). Therefore, the VS may be an immediate self-reward as the dolphin marks. The VS may also be an uninhibited emotional outburst as suggested by Dibble et al. (2016). The frontal lobe of the dolphin's brain is relatively small as is the limbic cortex (Morgane et al., 1980; Manger, 2006). Both of these brain areas are involved in the modulation of emotional behaviors in other mammals (e.g. Knight et al., 1999). Reduced action of these cortical regions has not been explicitly demonstrated experimentally in dolphins due to the technical and ethical difficulties of undertaking such a study. However, the mesolimbic dopaminergic reward pathway appears to be expanded in dolphins compared to other mammals (Manger et al., 2004), which together with the small prefrontal and cingulate cortex indicates that inhibition of emotional sounds may not have developed fully in dolphins. This may lead to the regular production of the VS by dolphins observed in this study and previous ones (Ridgway et al., 2014, 2015; Dibble et al., 2016). A limited cortical inhibition and expanded mesolimbic system might explain why dolphins appear to start producing a VS and then abruptly stop upon deciding to reject a fish (Ridgway et al., 2015). Dolphin Z produced a VS with every mark of a proud or moored mine simulator. Latching to the cable provided the dolphin with an immediate ‘gotcha’. On the other hand, dolphin Y produced the VS on 93% of marks for proud simulated mines. However, dolphin Y produced the VS only 44% of the time when marking near the buried target (Table 1). These buried mine simulators may have been more difficult. The lack of a VS on some of the marks by dolphin Y may suggest some immediate uncertainty about the successful completion of the task.

## CONCLUSION

Dolphins wore harness cameras during mine simulator tasks in the ocean. The camera record linked all dolphin sound with behavior. The timing of clicks varied in different phases of the task. During the detection phase, both dolphins produced continuous trains of clicks while searching. During the marking phase, both dolphins switched strategy. They used shorter click trains interrupted by periods of silence. Often after marking, but at other times as well, dolphins emitted short click packets. These packets were several clicks bounded by a period of silence. Since a dolphin must have a sense of the acoustic features of its clicks and how many clicks it produces, a packet may be a coded signal. The coded signal may be matched to an echo return, in essence, a matched filter. We suggest that packets were used to improve the signal to noise ratios for locating a boat or other distant target of interest. Victory squeals produced when marking the targets suggest to us that the dolphins know when they have succeeded in this multipart search and detection task.

## MATERIALS AND METHODS

We recorded video and sound from two female *T. truncatus* designated here as dolphin Y (age 34 years) and dolphin Z (age 15 years) as they searched, detected, and marked mine simulators. We equipped each dolphin's harness with a camera (GoPro Hero3+ or Garmin VIRB). The camera recorded video and sound with a bandwidth of 16 kHz. This acoustic bandwidth is narrower than the usual dolphin clicks, which may reach 150 kHz or more (Au, 1993). However, because the acoustic bandwidth of each click is so broad the camera system faithfully records the timing of each click. In a previous study, we made simultaneous recordings with a broadband hydrophone system and the same cameras used here. In that study, the timing of the dolphin's clicks was accurately represented by the camera, even though recording a limited 16 kHz bandwidth (Ridgway et al., 2015; Dibble et al., 2016).

Dolphin Y had the camera placed dorsally and sometimes laterally (Fig. 1C) on the harness; dolphin Z only wore the camera dorsally (Figs 1E and 2C). The cameras allowed the dolphin's head and/or rostrum to be visible as the animal swam and participated in mine simulator detection tasks. This allowed the instant the dolphin marked a target to be observed and thus could relate animal movements and behaviors to sound production. In the majority of recordings, if the camera was positioned dorsally, movements of the external nasal plug and blowhole were visible as the dolphin clicked, whistled, squealed or made simultaneous whistles and clicks (Ridgway et al., 2015).

As the animals search, operators on the boat observe their surface behavior and occasionally reward them with fish as they swim ahead of the boat searching the ocean for simulators. Searching is readily recognized as the dolphins make head scanning motions moving their rostrum from side to side and up and down. We were able to record two dolphins as they searched for targets in the open ocean. Dolphin Y swam with the operators' boat searching for mine simulators buried beneath the sediment or resting on the bottom (proud) at depths of 6-25 m. Dolphin Z searched both for proud simulators, but also for moored simulators anchored by a cable. Depths of the moored mine simulators ranged from 6 to 60 m. (Figs 1A and 2A) are schematic representations of the dolphins searching the ocean for sea floor mine simulators. When the dolphin found a target it returned to the boat, reported a positive detection and accepted a marker from the operator to identify the mine simulator's location (Fig. 1B,C,E). Fig. 2A represents dolphin Z searching the ocean for a moored simulator then after a positive detection, taking a marker and latching it to the mooring cable (Fig. 2B,C). All experiments were approved by the Animal Safety Committee of the U.S. Navy Marine Mammal Program, San Diego, CA, USA.

To identify and classify sounds, video recordings were displayed in Adobe Audition CS6 with Blackmann-Harris windows of 512 points. The recordings were then inspected aurally and visually.

## Supplementary Material

Supplementary information
