# Supplementary material for: Timing and context of dolphin clicks during and after mine simulator detection and marking in the open ocean
Source: Biol Open. 2018 Feb 15;7(2):bio031625. doi: 10.1242/bio.031625 (PMC5861363; doi:10.1242/bio.031625)
Supplement: Supplementary information [file biolopen-7-031625-s1.pdf]

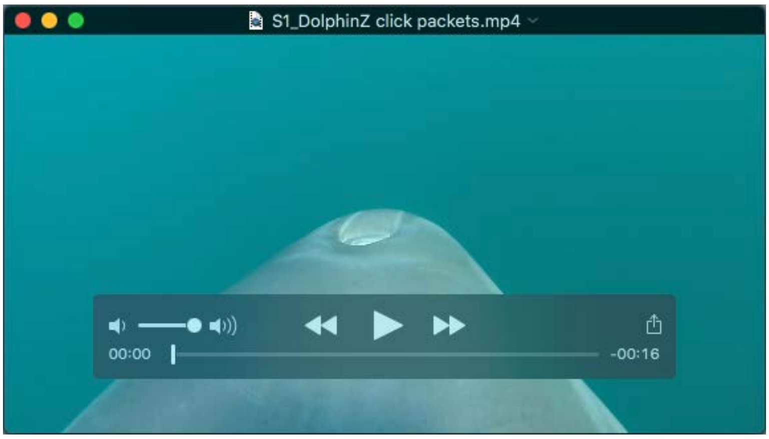

Movie 1: _DolphinZ Click Packets

Dolphin Z produces a click packet series (several individual click packets) ascending towards the boat after marking a bottom target. NOTE: boat becomes visible in top right FOV.

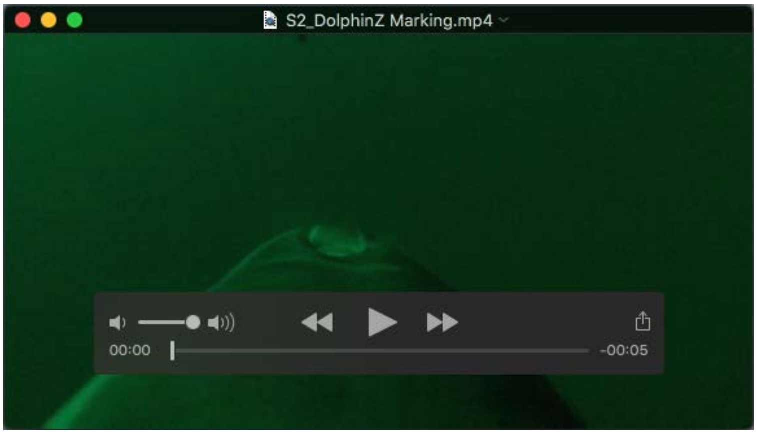

Movie 2: _DolphinZ Marking

Dolphin Z carries a marker to a moored simulator tethered to the sea floor. Before the marker contacts the mooring cable, dolphin Z begins producing a victory squeal lasting until after the marker appears to be secured to the cable.

Biology Open • Supplementary information
